# Diagnosis and Non-Surgical Treatment of Cranial Deformities in Children Under 2 Years of Age: Modern Methods and Approaches

**DOI:** 10.34763/jmotherandchild.20263001.d-25-00021

**Published:** 2026-02-10

**Authors:** Marat Rabandiyarov, Aleksandr Boiko, Anton Zolotukhin, Kirill Loviannikov, Azamat Zhailganov

**Affiliations:** Children’s City Clinical Hospital No. 2, Almaty, Republic of Kazakhstan; Saigak-Ortho Clinic, Almaty, Republic of Kazakhstan

**Keywords:** corrective helmets, 3D optical scanning, craniometry, non-invasive techniques, head shape

## Abstract

**Background:**

Cranial deformities in children under 2 can lead to functional and cosmetic issues, with their frequency increasing due to sleep positioning practices. This study aimed to evaluate the effectiveness of custom-made corrective helmets in treating these deformities.

**Material and methods:**

The study included 30 children under 2 in Astana, Kazakhstan, using 3D scanning to create custom corrective helmets worn for 23 hours a day over 5 months.

**Results:**

The results showed a significant improvement in the shape and symmetry of the skull after therapy. The asymmetry between the left and right front dimensions decreased from 9 mm to 3 mm, which corresponds to the age norm. The anterior-posterior size of the skull increased from 129 mm to 142 mm, the lateral size – from 123 mm to 129 mm, and the head circumference – from 41.5 cm to 43.5 cm, which indicates normal development of the skull. The majority of parents (90%) noted that children adapted easily to wearing helmets and experienced high levels of comfort. The data obtained indicate that the use of corrective helmets is an effective and safe method of non-surgical treatment of cranial deformities in young children. This avoids surgery and the associated risks, ensuring an improvement in the shape of the skull and the quality of life of patients.

**Conclusions:**

This study highlights the effectiveness of orthotic therapy for treating cranial deformities in young children, emphasizing the importance of early intervention and the potential for improved outcomes with timely, non-invasive treatment.

## 1. Introduction

Cranial deformities in young children (up to 2 years) are an urgent problem in modern paediatrics, neurosurgery, and paediatric neurology. These disorders, in particular positional (plagiocephaly – asymmetrical skull shape usually caused by pressure on the head at an early age) and premature closure of the skull sutures, which leads to deformation (craniosynostosis), can have both cosmetic and functional consequences. The incidence of cranial deformities in newborns is increasing, which is associated with both an overall increase in the survival rate of premature babies and the widespread implementation of recommendations for positioning infants to reduce the risk of sudden infant death syndrome. The study by S. Mussabekova et al. [1] examined the skulls of 187 men and 114 women from the two largest regions of Kazakhstan to assess the possibilities of forensic identification of the population of Kazakhstan by craniometric indicators. Significant differences in skull size were found depending on geographical and environmental conditions. The shape of the skulls in the studied regions is mostly brachycephalic, with a cranial index of more than 81% for men and 83% for women.

Thus, some studies on this topic, namely by A. Launonen et al. [2], investigated the difference in skull asymmetry between preterm and full-term infants. According to the results of the study, it was found that premature babies have more flattening of the back of the head than full-term ones, but the asymmetry gradually evens out in all groups. The disadvantage of such a study is the small sample size and the absence of cases with strong deformation, which limit the generality of the results.

E. Mercan et al. [3] investigated the features of skull growth in children with isolated side view (sagittal) craniosynostosis compared to children without abnormalities. It was found that the sagittal growth of the skull in children with craniosynostosis is significantly accelerated compared to the norm. The disadvantage is that the study does not consider the long-term effects of surgery on deformity correction. J. Foster et al. [4] investigated the influence of demographic factors on the development of cranial deformities. It was indicated that the difference in skull asymmetry depends on race, but not on gender or ethnic origin. However, cultural or environmental factors that can also influence the development of cranial deformities were not considered.

A. Grandoch et al. [5] studied the effectiveness of helmet deformity therapy at an early age and parental satisfaction. There was high parental satisfaction with this treatment method and a significant improvement in the shape of the skull after helmet therapy, but a small sample size limits the possibility of generalising the results to a wider population. In turn, D. Reshchikov et al. [6] studied the tactics of diagnosis and treatment of craniosynostosis in children after 2 years of age. According to this study, the best results are achieved in early surgery, and in cases of later treatment, reconstructive cranioplasty is possible. Other studies have assessed the prevalence of deforming shortened and broad skull shape due to early fusion of the coronal sutures (brachycephaly) using the cranial index. Thus, R. Ahluwalia et al. [7] noted that the prevalence of brachycephaly decreases with age, but is common in children under 2 years of age. A limitation of this study is the lack of details about the influence of environmental factors on the development of the skull.

Some studies have focused on the association between ultrasound cranial abnormalities and minor motor disorders in children. S. Demauro et al. [8] concluded that minor motor abnormalities were detected independently of cranial abnormalities on ultrasound, but led to increased medical resource requirements. The disadvantage is the lack of information about the long-term consequences of motor disorders. There were studies that evaluated the mechanisms of traumatic brain injuries in children under 10 years of age. According to T. Araki et al. [9], it was found that in children aged 0–5 years, injuries are most often caused by falls, while in children aged 6–10 years, injuries from road accidents are more common, requiring more intensive treatment. The study by I. Tamada et al. [10] was devoted to the analysis of technical aspects of skull reconstruction in children with increasing fractures. The study involved four children with growing fractures who needed surgery. Own bone grafts were used to reconstruct the skull, which ensured stable recovery of skull defects. The use of bone grafts not only restored the integrity of bone structures but also reduced the risk of rejection and ensured harmonious bone growth. No serious complications were detected in any patients after surgery, which indicates the effectiveness and safety of the method. However, the study was limited to a small sample of only 4 patients, so the results may not fully reflect the effectiveness of the method for a wider group of patients.

Thus, a large number of studies were focused on the treatment of skull deformities, but no research has been found on the diagnosis and treatment of such pathologies in children under 2 years of age, which necessitated the current study. The purpose of this study was to evaluate the effectiveness of therapy with corrective helmets for the treatment of skull deformities in young children. To achieve this goal, the following research tasks were performed: to analyse the causes and types of skull deformities; to review existing methods of diagnosis and therapy of skull deformities; to determine their advantages and disadvantages; to empirically investigate the effectiveness of diagnostics using three-dimensional photogrammetry and treatment with corrective helmets.

## 2. Materials and methods

The literature search was carried out in the electronic databases PubMed, Scopus, Web of Science, and Google Scholar. Combinations of keywords and MeSH terms were used: “cranial deformities”, “infants”, “craniosynostosis”, “plagiocephaly”, “brachycephaly”, “diagnosis”, “treatment”, “early childhood”. Search queries were generated using the logical operators AND and OR to refine the results.

Inclusion criteria: papers published mainly in the last 5 years, publications in English and Kazakh, studies containing information on the causes, types, methods of diagnosis and treatment of cranial deformities in children under 2 years of age, original studies, systematic reviews, meta-analyses. Exclusion criteria: papers related to adult patients or children over 2 years of age, studies with insufficient description of methodology or poor quality of evidence, duplicates, and non-peer-reviewed publications.

A total of 120 potentially relevant sources were identified. After reviewing the titles and annotations, 70 papers were selected for detailed analysis. After applying the inclusion and exclusion criteria, 34 literature sources were included in the final analysis. The content analysis method was used to organise and analyse the collected data. This approach helped to identify and classify the main causes and types of cranial deformities in young children and to determine modern approaches to their diagnosis and treatment.

During the initial assessment of infants, a thorough screening process was conducted to exclude any potential serious conditions, particularly craniosynostosis. Each child underwent a comprehensive examination, including a physical check-up and diagnostic methods such as 3D optical scanning. Special attention was paid to the nature of the cranial deformation, particularly any signs of premature suture fusion, which could indicate craniosynostosis. In cases where such a condition was suspected, the child was immediately excluded from the study. Thus, craniosynostosis was specifically excluded as part of the inclusion criteria, and only children diagnosed with positional plagiocephaly, brachycephaly, or similar conditions were selected for participation in the study. This approach ensured the accuracy and reliability of the sample, particularly in evaluating the effectiveness of non-surgical treatment methods for specific types of cranial deformities.

The severity of plagiocephaly was quantitatively assessed at the initial stage of the study using validated anthropometric indices, specifically the Cranial Vault Asymmetry Index (CVAI) and the cephalic index. These indices were employed to evaluate the degree of asymmetry and changes in head shape prior to the initiation of treatment. The inclusion of these anthropometric indices allowed for a standardised assessment of the deformities’ severity and provided a more accurate evaluation of the cranial condition in children at the start of therapy. Determining the baseline severity of the deformities is crucial for understanding the treatment’s effectiveness, as the degree of initial deformity can significantly impact the results and duration of therapy. The data collected regarding the baseline severity of plagiocephaly, based on the aforementioned indices, were used to assess the influence of the initial condition on the final treatment outcomes.

A subgroup analysis was conducted based on the severity of cranial deformities and the age at the start of therapy. This allowed for a more detailed interpretation of the results and provided insights into whether the favourable outcomes were consistent across the entire spectrum of deformities or predominantly observed in milder cases and younger patients. For severity-based stratification, patients were divided into two groups: those with mild deformities and those with moderate to severe deformities. The results indicated that more significant improvements in cranial symmetry were observed in the group with milder deformities, where the therapeutic effect was achieved more quickly and with shorter treatment durations. In contrast, children with more severe deformities required longer periods of therapy and showed relatively smaller improvements, suggesting that the degree of initial deformity can influence the effectiveness of orthotic therapy.

When analysing the impact of age at the start of therapy, younger patients (those who began therapy before 6 months of age) demonstrated greater improvements in head shape and required less time for correction. This aligns with the known age-dependent cranial remodelling potential, where younger children exhibit greater malleability of cranial bones, leading to more pronounced and quicker results. Older children, on the other hand, showed slower progress and required longer treatment durations to achieve similar outcomes, emphasising the importance of early intervention.

When analysing diagnostic methods, a comparative analysis of modern diagnostic technologies was carried out, including 3D computed tomography with a low radiation dose, EOS imaging, functional magnetic resonance imaging, diffusion tensor imaging, three-dimensional ultrasound, optical 3D scanning, virtual surgical planning and 3D printing, stereophotogrammetry, ultrasound holography, and 4D visualisation. The characteristics of each method were summarised in a table to visually compare their advantages and limitations.

Similarly, non-surgical approaches such as cranial remodelling orthotics, positional therapy, physical therapy (kinesiotherapy), 3D printed orthoses, dynamic orthotics, non-invasive laser therapy, kinesiotaping, electrical muscle stimulation and biofeedback therapy were reviewed for treatment methods. Indications, efficacy, limitations, and features of use in young children were analysed for each method.

Statistical data and results of clinical studies from selected sources were used in the preparation of tables and graphic materials. This allowed summarising the information and presenting it in an easy-to-analyse format. The results obtained were critically evaluated considering the level of evidence and quality of research. The interpretation considered statistical indicators of the effectiveness of the methods, clinical significance for patients, and practical aspects of application in real conditions. Special attention was paid to the safety of methods for young children and possible side effects.

An empirical study of the effectiveness of treatment of cranial deformities using corrective helmets involved 30 children under 2 years of age, mainly up to 1.5 years, since the study lasted 5 months and was aimed at evaluating the effectiveness of treatment in young children. The study was conducted in the city of Astana (Kazakhstan) at one of the children’s hospitals, which remains confidential for ethical reasons.

Criteria for inclusion in the sample: children aged 3 to 24 months with a diagnosed cranial deformity (plagiocephaly, brachycephaly, etc.), no serious comorbidities or congenital abnormalities that may affect the growth and development of the skull, consent of parents or legal representatives to the child’s participation in the study, the use of corrective helmets, and the possibility of regular visits to the doctor to monitor and correct treatment. Exclusion criteria from the sample: children older than 24 months or younger than 3 months, the presence of craniosynostosis or other pathologies requiring surgery, serious neurological, genetic or metabolic diseases, parents’ refusal to participate in the study or failure to follow the treatment protocol. The sample was not random and was formed based on the availability principle. Patients were selected from children who went to the hospital with a diagnosis of skull deformity between January and June 2024. Parents were asked to participate in the study after learning more about its purpose, methods, and possible risks.

At the beginning of the study, each child underwent a comprehensive diagnosis to assess the degree and type of skull deformity. The main diagnostic method was 3D optical scanning, which made it possible to obtain high-precision three-dimensional images of the skull without using ionising radiation. This method is completely non-invasive and safe for children, which is especially important in paediatric practice. The resulting 3D images were stored digitally for further analysis and comparison of treatment dynamics. Some parents have agreed to provide these images and photos before and after treatment for scientific purposes; they are presented in Appendix A and Appendix B.

After diagnosis, a correction helmet was individually made for each child based on the resulting 3D model of the skull. The helmet was modelled in such a way as to create light pressure on the protruding areas of the skull and provide space for growth in flattened areas, contributing to the gradual correction of the shape of the head. Children wore helmets for 23 hours a day, taking them off only for an hour for hygiene procedures. The duration of treatment was 5 months. Regular follow-up examinations were conducted every two weeks to monitor progress and, if necessary, adjust the internal shape of the helmet according to the dynamics of skull growth, as well as individually, at the request of parents in case of complaints or other reasons.

Equipment used: optical 3D scanner (manufacturer: China), which provides high resolution and accuracy of measurements, without the use of radiation. Corrective helmets made of hypoallergenic materials that meet international safety standards for children’s products. The design of the helmet provided ventilation holes and the ability to adjust to ensure the comfort of the child.

To assess the effectiveness of treatment, craniometric parameters were measured before and after therapy, in particular: anterior-posterior skull size, lateral skull size, left front size, right front size, and head circumference. The obtained data were analysed using statistical methods, in particular, the average values were calculated.

The study was conducted in compliance with the ethical norms and principles of the Helsinki Declaration [11]. Each child’s parents or legal representatives have signed informed consent to participate in the study and use of personal data. The name of the medical facility remains confidential in order to protect patients’ privacy and comply with ethical standards.

During regular check-ups, parents were asked to answer a short assessment questionnaire (Table 1).

**Table 1.** Questionnaire text

**No.**	**Question**	**Answers**
1.	How easily did your child adapt to wearing a helmet, and did you have any difficulties caring for them?	Very easy, there were no difficulties
		It took some time to adapt, but there were no problems
		The adaptation was difficult, but we managed
		There were many difficulties, the child never got used to it
2.	Do you feel an improvement in the shape of your child’s head from the very beginning of using the helmet?	Noticeable improvement, the shape of the head has become more symmetrical
		Moderate improvement, positive changes are observed
		Minor improvement, but still hard to notice the result
		No improvements are noticeable, and the shape of the head has hardly changed
3.	How comfortable is it for a child to wear a helmet in everyday life (considering sleep time, eating, and activity)?	Very comfortable, the helmet does not interfere with the child
		Quite comfortable, but sometimes there is discomfort
		It is not always comfortable, but the child gets used to it
		The child is uncomfortable, the helmet interferes with everyday life

*Source:* compiled by the authors.

Parents’ responses helped to supplement objective data with a subjective assessment of the effectiveness and tolerability of treatment. The results obtained were analysed based on the age norms of skull development in children. Special attention was paid to the dynamics of changes in craniometric parameters and the reduction of asymmetry between the left and right sides of the skull. Subjective feedback from parents regarding the comfort and effectiveness of therapy was also taken into consideration.

## 3. Results

### 3.1. Causes and types of skull deformities in young children

Skull deformity is a structural change in the shape or size of the skull bones that can be caused by external or internal factors. It often occurs due to constant pressure on certain areas of the skull or due to impaired bone development. In children under two years of age, skull deformity often occurs due to a number of physical, physiological, and external factors that affect the flexibility of the skull bones. Since the sutures and fontanelles on the skull have not yet grown, and the bones remain soft, any prolonged or excessive pressure on the skull can lead to deformities. An important factor is the position in which the child spends most of their time: for example, when the newborn often lies on its back, this creates constant pressure on the occipital part of the skull, which can change its shape.

The uniform development of bone tissue can also be disrupted due to muscle tone. Infants with insufficient control of the neck and back muscles may experience a situation where the child cannot change the position of the head evenly, which leads to excessive pressure on one of its parts. Muscle weakness or asymmetry in tone, for example, in the case of congenital torticollis, also contributes to an uneven distribution of pressure.

Another factor is the state of intrauterine development. If the foetus is in a restricted position during pregnancy or exerts excessive pressure on the skull due to the narrowness of the uterus, incorrect position, or multiple pregnancies, this can affect the shape of the infant’s skull even before birth. There is also the influence of hormonal factors that affect the development of bone tissue. In particular, the levels of calcium and other minerals in the mother and child play an important role in bone strength, and their deficiency can reduce the density of bone structures in the skull, making it more susceptible to deformities.

External mechanical factors after birth also include prolonged exposure to car seats, baby strollers, or cribs, which can restrict natural head movements. Inappropriate care methods, such as not spending enough time on the child’s stomach, can also reduce the overall strength of the neck muscles, which increases the risk of deformities.

Types of skull deformities include both natural and artificial shape changes that can occur due to various factors. Artificial deformity of the skull, or so-called “artificial craniality”, is a modification of its shape, carried out intentionally. For this purpose, special devices are used, superimposed on the child’s head at an early age, gradually changing the shape of the skull. This type of deformity has been used in many cultures and has remained a subject of interest for anthropologists and researchers of human morphology, as it affects the modularity and overall integration of skull structures [12].

Craniosynostosis is a pathological condition in which one or more cranial sutures heal prematurely, which prevents the normal growth of the skull and leads to the development of a characteristic deformity. Depending on which suture has undergone premature fusion, the skull takes on a certain shape. For example, if the sagittal suture grows together, an elongated deformity called “scaphocephaly” occurs, and when the coronal sutures grow together, a wide and short skull structure is formed. Craniosynostosis is both an aesthetic and functional problem that requires surgical intervention to improve the shape and prevent complications [13, 14].

Positional deformity of the skull, or plagiocephaly, occurs due to constant pressure on one side of the head, often associated with the infant’s sleeping position. It causes unilateral flattening of the skull and is quite common among young children, especially after the recommendations of doctors to put the child to sleep on his back to prevent sudden infant death syndrome. Positional plagiocephaly can be corrected using positioning techniques or special orthopaedic helmets that gradually correct the shape of the skull [15]. Brachycephaly is also a positional deformity in which the skull becomes short and wide. This is conditioned by a restriction in expansion in the anteroposterior direction. It often occurs because the child is constantly lying on their back. Correction of this deformity depends on the severity, and in some cases a special corrective helmet is used to achieve a more symmetrical skull shape [16].

Crouzon syndrome is a genetic disorder characterised by premature fusion of the cranial sutures, which causes abnormalities in the development of the skull and face. Patients with this syndrome have a flat middle part of the face, hypoplasia of the orbits leading to exophthalmos, and signs such as a broad front view (frontal) part and nasal deformity [17]. Apert syndrome is a rare congenital disorder associated with premature fusion of the cranial sutures, which is accompanied by severe finger fusion (syndactyly) on the hands and feet. It is caused by mutations in the *FGFR2* or *FGFR3* genes, which lead to facial abnormalities, in particular, hypoplasia of the middle part of the face and specific dental and maxillary abnormalities [18].

Deforming plagiocephaly is a curvature of the shape of the skull caused by external pressure on the child’s head after birth. This is one of the most common disorders of the skull shape in children, which is associated with the risk of motor development disorders. To correct the shape of the skull, a special orthopaedic helmet can be used, which improves the appearance and motor skills of the child [19]. Trigonocephaly is characterised by a triangular head shape that occurs due to premature fusion of the metopic suture, which causes a wedge-shaped forehead and narrowing of the anterolateral part of the head. In children with this deformity, there may be an increase in intracranial pressure, which can affect the development of the nervous system. Treatment for trigonocephaly involves cranioplasty, which is usually performed before the child reaches one year of age [20].

Thus, cranial deformities can occur both for natural causes associated with developmental pathologies, and as a result of artificial exposure chosen for cultural or therapeutic reasons. Each type of deformity requires an individual approach to diagnosis and treatment.

### 3.2. Comparative review of modern methods of diagnosis and treatment of cranial deformities in young children

Cranial deformities in young children are a complex medical problem that requires modern diagnostic approaches and effective treatment methods. Abnormal skull shapes can be both congenital and acquired in nature and often lead to functional disorders, such as developmental delay or cognitive impairment. Early detection of these pathologies is important to ensure proper development of the child, and modern methods of diagnosis and treatment can reduce the risk of negative consequences.

In recent years, significant progress has been made in the diagnosis and treatment of cranial deformities in children. There are many diagnostic approaches, from visual assessment to high-tech techniques such as computed tomography and magnetic resonance imaging. Therapeutic methods have also expanded significantly: along with conservative approaches, such as the use of orthopaedic helmets, surgical correction methods are actively used.

The purpose of this section is to conduct a comparative review of modern methods of diagnosis and treatment of cranial deformities in young children. The importance of analysing different approaches lies in determining their effectiveness, safety, and availability, which are key factors in choosing the right method for a particular patient. Table 2 shows a list of modern methods for diagnosing cranial deformities and their characteristics.

**Table 2.** Modern methods for diagnosing cranial deformities

**Method name**	**Characteristics**
3D computed tomography with low radiation dose	Provides high-quality three-dimensional images of the skull with minimal radiation exposure. Allows assessing bone structures in detail and planning surgical intervention for complex deformities.
EOS-imaging	Innovative system for obtaining two- and three-dimensional images in an upright position of the patient using ultra-low radiation doses. Allows simultaneously assessing deformities of the skull and spine
Functional magnetic resonance imaging	Displays brain activity in real time, helping to assess the impact of skull deformities on functional areas of the brain. Useful for treatment planning that preserves important neural pathways.
Diffusion tensor imaging	Specialised type of magnetic resonance imaging (MRI) that visualises the pathways of the brain’s white matter. Helps to identify possible violations of neuronal connections due to deformities of the skull.
Three-dimensional ultrasound examination	Non-invasive radiation-free method that creates a 3D image of the skull. Ideal for diagnosis in infants and young children with soft bones.
Optical 3D scanning	Uses light technologies to create high-precision three-dimensional models of the skull surface. Fast and safe method for evaluating external deformations.
Virtual surgical planning and 3D printing	Uses light technologies to create high-precision three-dimensional models of the skull surface. Fast and safe method for evaluating external deformations.
Stereophotogrammetry	Technology for obtaining three-dimensional images using photos from different angles. Allows non-contact assessment of the shape and symmetry of the skull.
Ultrasonic holography	New method that uses ultrasound waves to create holographic images of the internal structures of the skull. Provides a safe alternative to X-rays for diagnosis.
4D visualisation	Combines three-dimensional images with time measurement, helping to observe dynamic changes in the skull during movement or growth. It is useful for studying the functional aspects of deformations.

*Source:* compiled by the authors based on L.M. Pogliani et al. [21], A. Neverauskienė et al. [22], J. Vazquez et al. [23].

The latest methods for diagnosing cranial deformities are characterised by a significant variety of technical approaches and capabilities, allowing the selection of the optimal tool depending on the specifics of the case and the patient’s needs. In particular, low-dose 3D computed tomography is an important method for obtaining high-quality images of bone structures. This study allows detailed planning of surgical interventions for complex deformities, reducing radiation exposure and ensuring maximum accuracy. However, even when the radiation dose is reduced, this method still involves a certain level of radiation exposure, which may limit its use, especially for children or patients who need multiple repeated examinations.

The innovative EOS imaging method also provides high-quality images with a very low radiation dose, but allows performing diagnostics in an upright position, which allows simultaneously assessing both deformities of the skull and spine. This is especially useful for patients with complex postural disorders or for those who need a comprehensive approach to the treatment of craniocerebral abnormalities. EOS imaging, however, has some limitations in terms of the accuracy of mapping the bone structures of the skull compared to 3D CT, which may sometimes require supplementation with other methods.

Functional magnetic resonance imaging is a unique method because it provides real-time information about brain activity, helping to assess how cranial deformities can affect functional areas of the brain. This is especially important for planning surgery, when it is necessary to preserve key neural connections and not disrupt vital functions. However, functional magnetic resonance imaging (fMRI) is a technologically complex and expensive procedure that requires special equipment and qualified personnel, which can increase its cost and limit its availability for widespread use.

Another method from the MRI group is diffusion tensor imaging, which focuses on visualising the white matter pathways of the brain and can detect potential neural connection disorders caused by cranial deformities. Diffusion tensor imaging (DTI) provides an opportunity to analyse neuronal connections in detail and can be indispensable for patients with disorders affecting the nervous system. However, like fMRI, DTI is quite an expensive method and requires special equipment, which can limit its use.

Three-dimensional ultrasound provides radiation-free images of the skull and is non-invasive, making it ideal for diagnosis in infants and young children with soft bones. This is a safe method that can be repeated without risk to health. However, this method is less effective for adults, as denser bones make it difficult to get a clear image.

Optical 3D scanning, which uses light technology to create accurate three-dimensional models of the skull surface, is a fast and safe method for assessing external deformations. Its main advantage is that the method does not require a contact examination and is completely devoid of radiation exposure. However, this approach is limited to analysing only external structures, and its capabilities are insufficient to assess internal changes in the skull bones, which is sometimes important for accurate diagnosis.

As for virtual surgical planning and 3D printing, this is a modern approach that combines computer modelling and 3D printing technologies for the manufacture of individual implants and detailed planning of surgical interventions. This method provides high accuracy during operations and reduces their duration, but it requires significant financial costs and preparation time, which can complicate its implementation in clinical practice.

Stereophotogrammetry is a non-contact method that uses photographs from different angles to create three-dimensional images, helping to assess the shape and symmetry of the skull without direct contact with the patient. This is a safe and fast method of visualisation, but like optical 3D scanning, it is limited to analysing external characteristics. Among other new methods, it is worth noting ultrasound holography, which uses ultrasound waves to create holographic images of the internal structures of the skull. This method has significant potential as a safe alternative to X-ray examinations, but it is still limited in detail and requires further research and improvement to produce more accurate images.

Ultimately, 4D imaging, which combines three-dimensional imaging with time measurement, allows observing dynamic changes in the skull during movement or growth, which is important for studying the functional aspects of deformities. This method allows assessing how the skull structure changes in real time, which is useful for determining the effect of deformities on the patient’s functional capabilities. However, due to the complexity and high cost of the equipment, its use is limited to research centres and specialised clinics.

Thus, each of these methods has its own unique advantages and disadvantages, which determine the feasibility of their use depending on specific clinical needs, patient age, complexity of pathology, and availability of resources. Table 3 shows the latest methods of treating cranial deformities and their characteristics.

**Table 3.** Methods of treatment of cranial deformities and their characteristics

**Method of non-surgical treatment of skull deformity**	**Characteristics**
Corrective helmet	The use of specially made helmets that gradually correct the shape of the infant’s skull. It is effective in the treatment of positional plagiocephaly, brachycephaly, and scoliocephaly. The method consists of directing the growth of the skull bones by lightly pressing on the protruding areas and creating space in the flattened regions to allow natural symmetrical growth.
Positional therapy	A non-constructive intervention that involves changing the position of the child’s head during sleep and activity to reduce pressure on flattened areas. It is effective for mild forms of positional plagiocephaly. It includes recommendations for lying on the stomach under supervision, changing the position of the crib, and stimulating head turns.
Physical therapy	Using 3D printing technology to create individual orthopaedic devices that correct the shape of the skull. Provides precise compliance with the patient’s anatomy and comfort in use. It is effective for various types of deformities, in particular, plagiocephaly and brachycephaly.
Dynamic helmet (orthosis) therapy	The use of dynamic orthoses that adapt to the child’s growth and provide a constant corrective effect. It is effective in the treatment of asymmetric deformities. The method allows controlling the correction process and reduces the need for frequent replacement of orthoses.
Non-invasive laser therapy	The use of low-intensity laser radiation to stimulate cellular metabolism and improve blood circulation in areas of deformity. It can help to accelerate the natural correction of the shape of the skull in case of mild deformities.
Kinesiotaping	Application of special elastic tapes to the scalp and neck to correct muscle tone and head position. It is used as an auxiliary method for mild deformities or in combination with physical therapy. Helps to improve the symmetry of movements and head position.
Electrical muscle stimulation	The use of electrical impulses to stimulate the muscles of the neck and shoulder girdle to correct positional deformities. It is useful for muscle weakness or an imbalance that leads to skull asymmetry. It is carried out under the supervision of a specialist in physical rehabilitation.
Biofeedback therapy	The use of biofeedback technologies to train the patient to control muscle activity. Helps in correcting the position of the head and neck, is effective for functional deformities. The method is non-invasive and safe, suitable for older children.

*Source:* compiled by the authors based on C.E. Ripley et al. [24], T. Graham et al. [25], J. Terpenning [26].

Methods of non-surgical treatment of cranial deformities, such as cranial remodelling orthotics, positional therapy, physical therapy, 3D-printed orthoses, dynamic orthosis therapy, laser therapy, kinesiotaping, electrical muscle stimulation, and biofeedback therapy, offer a multifaceted approach to the correction of craniofacial deformities in young children. Each method has its own advantages and limitations, which depend on the type of deformation, the degree of complexity and individual needs of the child.

Cranial remodelling orthotics using corrective helmets are effective for treating severe forms of positional plagiocephaly, brachycephaly, and scoliocephaly. This approach allows gradually correcting the shape of the skull due to light pressure on convex areas and creating conditions for growth in flattened areas. However, this method requires regular supervision by a specialist and significant financial costs, which can become an obstacle for families. Moreover, the child must always wear a helmet, which can be psychologically and physically uncomfortable. Positional therapy is a less invasive approach that uses changing the position of the child’s head to reduce pressure on flattened areas. This is a simple, economical, and safe method for mild forms of deformities that can be practised at home under the supervision of parents. However, its effectiveness is limited, especially with significant deformations, and it takes a long time to achieve a visible result. Positional therapy may be less effective for children who have already developed a significant deformity.

Physical therapy, in particular kinesiotherapy, helps to correct muscle tone and improve the child’s motor skills. This is especially true for torticollis, which often accompanies deformities of the skull. Physical exercises for the neck and shoulder girdle help to reduce asymmetry and improve overall mobility, although the effectiveness of this method depends on the regularity of training and the experience of the specialist performing the therapy. 3D-printed orthoses provide the ability to create individually customised devices that exactly match the child’s anatomy. They are easy to use and provide high correction accuracy. This method is innovative and potentially more affordable in the future due to the reduced cost of 3D printing. However, it is still technologically dependent and requires a certain level of availability of high-tech equipment.

Dynamic orthosis therapy allows adapting the device to the child’s growth, which reduces the need for frequent replacement and allows maintaining the corrective effect for a long time. This approach is convenient and cost-effective, but efficiency can be reduced in cases of complex deformations that require more effort to correct. Non-invasive laser therapy, based on stimulating cellular metabolism and improving blood circulation, is useful for mild forms of deformities. It can speed up natural correction and is safe, but has limited effectiveness for more serious deformities. This method is not yet generally accepted and requires additional research to determine its long-term effectiveness.

Kinesiotaping, which involves applying elastic tapes to correct muscle tone, helps to maintain a symmetrical head position and is an additional method to physical therapy. It is easy to use, but the effectiveness depends on the correct technique of applying tapes, which requires an experienced specialist.

Electrical stimulation of the neck and shoulder girdle muscles provides an additional corrective effect for muscle weakness, which helps to restore symmetry. This method requires professional supervision and can cause discomfort in children. Biofeedback therapy is safe and non-invasive, helping the child learn to control muscle activity, which is especially useful for older children. However, it requires a certain level of consciousness from the child, so its use is limited in children under two years of age.

Therefore, non-surgical methods of treating cranial deformity at an early age can be effective, but their choice should be based on the individual characteristics of the child, the degree of deformity and the financial capabilities of the family. Because early intervention is key, a combined approach involving multiple techniques is often the most effective, allowing for the best results without the need for surgery.

### 3.3. Empirical study of the effectiveness of treatment of cranial deformities using corrective helmets

At the beginning of this study, each child underwent a comprehensive diagnosis to assess the extent of skull defects. The main purpose of this procedure was to determine the degree of abnormalities in the structure of the skull bones, which is crucial for an accurate understanding of the anatomical features and abnormalities in each child. For this survey, the latest method was chosen – optical 3D scanning, which provides the most detailed image of the object and allows for the detection of even minor changes or defects.

The use of 3D scanning to study skull defects in childhood was a real breakthrough. One of the main advantages of the method is its absolute non-invasiveness, which means no pain or discomfort for the patient. Children do not experience physical or psychological difficulties during the procedure, which makes diagnostics convenient and, most importantly, safe. In addition, the method does not use ionising radiation, which is potentially dangerous for the body, especially for children. Therefore, optical 3D scanning is ideal for surveys where a safe approach that can be repeated repeatedly is important. In addition to safety, the 3D scanning method also provides high accuracy of results, which is important for detecting the smallest defects that could go unnoticed when using conventional examination methods such as X-rays or MRI. The resulting images have a high resolution and allow forming a three-dimensional model of the skull, which reflects in detail the structure of bones, their shape, size, and possible deformations. This model gives doctors and researchers the opportunity to conduct a thorough analysis and objectively assess the situation. Another important feature of this method is the ability to store all received data in digital format. This helps to record the initial indicators for each patient, which allows conducting long-term monitoring of changes. If necessary, the latest results can be easily compared with the previous ones, assessing the effectiveness of treatment or the development of certain deviations over time. With this method, doctors can develop more personalised treatment plans tailored to the patient’s specific needs.

Overall, the choice of optical 3D scanning as the main diagnostic method in this study was conditioned by a combination of its accuracy, safety, and convenience, which is especially important for working with children. As a result of applying this method, it was possible to collect valuable data on the anatomical features of the skull in children, which is an important basis for further stages of research and development of appropriate therapeutic measures aimed at eliminating the detected defects or minimising their impact on the child’s development. The resulting images of several respondents are shown in Appendix A.

Presented 3D reconstructions of the skull of young children show different projections of the skull: top view (axial), sagittal, and frontal, which provides a comprehensive assessment of its morphology. The use of colour mapping shows the depth or degree of deviation of the surface from the reference plane, which is useful for clearer visualisation of asymmetries and deformations. The polygonal grid on the surface of the 3D model indicates the use of the triangulation method for surface reconstruction and allows quantitative measurements of craniometric parameters. Various imaging options (with a smooth surface and with a polygonal grid, with or without colour mapping) are designed to comprehensively analyse the shape of the skull and identify potential craniosynostosis, plagiocephaly, brachycephaly, scaphocephaly, and other craniofacial abnormalities. 3D models of skulls are designed to provide an objective assessment of its morphology and dynamics of the process in cases where the clinical picture is not sufficiently pronounced for visual examination. In this way, it was also possible to measure some parameters, such as anterior-posterior skull size, lateral skull size, left anterior size, right anterior size and head circumference to better illustrate the dynamics of changes, which are presented in Table 4.

**Table 4.** Initial measurements of skull parameters (average values)

**Area**	**Value**
Anterior-posterior skull size (AP), mm	129
Lateral, mm	123
Right anterior size (RA), mm	136
Left anterior size (LA), mm	127
Circumference, cm	41.5

*Source:* compiled by the authors.

Establishing standard indicators of craniometric parameters in children of the first two years of life is difficult due to the high dynamics of their growth. In general, in children under 2 years of age, AP usually increases gradually. In newborns, this size is approximately 95–110 mm and gradually increases to about 120–140 mm at the age of 2 years. It is important to consider the individual growth rate, so the size may vary slightly.

The lateral size or width of the skull in children under 2 years of age also gradually increases. On average, this parameter is 85–95 mm in newborns and can reach 120–130 mm by the age of 2 years. LA and RA are usually examined to detect skull asymmetry, although the exact norm values may vary depending on clinical data, as these measurements are more individual. Normally, both indicators should be close in size and be about half of the anteroposterior size (about 50–70 mm in children under 2 years of age). A difference of more than 5 mm between the left and right dimensions may indicate asymmetry.

Head circumference is an important parameter for monitoring the overall development of the skull. On average, the length of the head circumference in newborns is about 34–36 cm. By 6 months, it can grow up to 42–44 cm, and by 2 years – reach 48–50 cm, this is one of the main parameters indicating the overall development of the brain and skull [27, 28].

A comparison was made between subgroups of children who began therapy before 6 months and after 6 months. The results showed that children who started treatment at an earlier age (before 6 months) demonstrated significantly greater improvements in head shape compared to those who started therapy later. This aligns with established data indicating that age is a crucial factor influencing the cranial remodeling potential. Younger patients achieved faster and more substantial improvements in head shape, which also allowed for a shorter treatment duration. Specifically, children who began wearing corrective helmets before 6 months required less time to achieve the desired result compared to those who started treatment at an older age. This highlights the importance of early intervention for optimal treatment outcomes, as the cranial remodeling capacity decreases with age.

The potential for cranial remodeling is known to be highly age-dependent, with younger children exhibiting a greater ability to modify the shape of their skulls. This is due to the increased flexibility and malleability of the cranial bones in early infancy, which allows for more significant and quicker changes in head shape when non-surgical treatment methods, such as corrective helmets, are applied. As the child grows, the sutures in the skull begin to fuse and the bones harden, reducing the cranial flexibility and, consequently, the effectiveness of treatments like helmet therapy.

The timing of treatment is crucial, as initiating therapy at a younger age typically leads to faster and more substantial improvements in head shape. Children who begin treatment before 6 months of age tend to require shorter durations of therapy, as the bones are more responsive to the corrective forces exerted by the helmets. In contrast, older children may need extended periods of treatment to achieve similar results, as their cranial bones are less pliable and more resistant to change. Therefore, early intervention is key in achieving optimal outcomes, as it allows for more efficient use of the therapeutic period and enhances the chances of correcting the deformities before the skull becomes less adaptable to changes.

Given the information provided on the dynamics of skull growth in children under two years of age, the values of AP and lateral size are within the age norm. However, there is an asymmetry between LA and RA, with a difference of 9 mm exceeding the threshold value of 5 mm, which indicates the existing deformation in most respondents. The value of the head circumference is slightly lower than the average for children of 6 months, although considering individual variability, it is impossible to unequivocally state deviations from the norm without additional data, such as the age of the child and other anthropometric indicators.

Treatment of cranial deformities in children was carried out using individually made corrective helmets. The choice of corrective helmets for the treatment of cranial deformities in young children is conditioned by a number of advantages of this method. Helmet therapy is a non-invasive correction method that avoids surgical intervention and associated risks. Individual production of helmets based on 3D models of the head provides an accurate and personalised approach to treatment, considering the characteristics of each patient’s deformity. The constant but gentle pressure exerted by the helmet on certain areas of the skull effectively directs its growth, helping to correct the shape without discomfort for the child. The high effectiveness of helmet therapy has been confirmed by numerous clinical studies. In addition, this method is relatively easy to use, well tolerated by patients and allows achieving significant results in a relatively short time. Considering all these factors, corrective helmets are the optimal choice for treating cranial deformities in young children, providing safe, effective and comfortable correction.

Thus, the helmet was made based on a 3D model of the child’s head obtained by scanning. The inner surface of the helmet was modelled in such a way as to create pressure on the protruding areas of the skull and provide space for growth in the sinking zones. This directed the growth of the skull in the desired direction and gradually correct the deformity. Fixing the helmet on the child’s head was carried out using special belts and fasteners. The design of the helmet provided holes for ventilation and comfortable wearing. Regular check-ups with a doctor (every 1–2 months) helped to monitor the correction process and, if necessary, adjust the internal shape of the helmet in accordance with the dynamics of skull growth. Children wore helmets for 23 hours a day, taking them off only for an hour for hygiene procedures. The duration of treatment was 5 months.

In addition, during one of the regular examinations, parents were asked questions to assess the subjective perception of the effectiveness of treatment and the comfort of the child. Collecting such information allowed for a more complete picture of the course of therapy and considering the individual characteristics of each patient. The question of adapting to wearing a helmet and the difficulty of caring for it helps to understand how easily the child and parents have integrated the helmet into everyday life, and identify potential problems associated with its use. The question of noticeable improvements in the shape of the head allows evaluating the effectiveness of treatment from the standpoint of parents who constantly monitor the child. The subjective assessment of parents is an important addition to the objective data obtained by 3D scanning. The question of the child’s comfort when wearing a helmet helps to identify potential inconveniences and prevent the development of skin irritations or other negative consequences. Focusing on the child’s comfort during sleep, eating, and activity allows us to assess the impact of the helmet on various aspects of their daily life. The information obtained allows adjusting the mode of wearing the helmet or making changes to its design to increase the comfort of the child. Parents’ responses are shown in Table 5.

**Table 5.** Parents’ responses regarding the comfort and effectiveness of corrective helmets

**Question 1: How easily did your child adapt to wearing a helmet, and did you have any difficulties caring for them?**	

Very easy, there were no difficulties	21 people
It took some time to adapt, but there were no problems	7 people
The adaptation was difficult, but we managed	2 people
There were many difficulties, the child never got used to it	0 people

**Question 2: Do you feel an improvement in the shape of your child’s head from the very beginning of using the helmet?**	

Noticeable improvement, the shape of the head has become more symmetrical	18 people
Moderate improvement, positive changes are observed	10 people
Minor improvement, but still hard to notice the result	2 people
No improvements are noticeable, and the shape of the head has hardly changed	0 people

**Question 3: How comfortable is it for a child to wear a helmet in everyday life (considering sleep time, eating, and activity)?**	

Very comfortable, the helmet does not interfere with the child	15 people
Quite comfortable, but sometimes there is discomfort	12 people
It is not always comfortable, but the child gets used to it	3 people
The child is uncomfortable, the helmet interferes with everyday life	0 people

*Source:* compiled by the authors.

Analysis of the results of a survey of 30 parents provides valuable information about the effectiveness, safety and tolerability of this method of therapy. Positive feedback from parents confirms the clinical effectiveness of helmets and their ability to correct the shape of the skull in young children. The easy adaptation to wearing a helmet, which was reported by 67% of parents, indicates a well-thought-out design of helmets and their ergonomics. This minimises stress for both the child and parents, contributing to a positive perception of treatment. It is important to note that even in cases where adaptation took longer (27%), parents successfully overcame the initial difficulties, which underscores the importance of supporting and advising specialists at the initial stage of therapy.

A high percentage of parents (60%) who reported a marked improvement in skull symmetry is a key indicator of the effectiveness of helmet therapy. This suggests that the soft but constant pressure exerted by the helmet actually directs the growth of the skull bones in the right direction. The moderate improvement observed in 33% of children can be explained by the individual rate of development and the degree of severity of the initial deformity. In such cases, it is important to continue monitoring and adjust the treatment plan if necessary. A small number of parents (7%) who have not yet noticed significant changes emphasise the importance of realistic expectations and understanding that correction of skull deformity is a gradual process.

The high level of comfort when wearing a helmet, noted by 90% of parents, is an important advantage of this method of treatment. This contributes to good tolerance of the helmet by the child and minimises the risk of side effects such as skin irritation. In general, the results of the survey show that helmet therapy is an effective, safe, and convenient method of treating cranial deformities in young children. However, it is important to keep in mind the need for an individual approach to each patient, regular monitoring, and correction of the treatment plan if necessary.

After completing a five-month course of daily treatment with corrective helmets, repeated measurements of the patients’ cranial parameters were performed. Detailed results of these measurements are presented in Table 6. To clearly demonstrate the changes that occurred in the shape of the skull during treatment, comparative images are shown in Appendix B.

**Table 6.** Final measurements of skull parameters (average values)

**Area**	**Value**
AP, mm	142
Side, mm	129
RA, mm	139
LA, mm	136
Circumference, cm	43.5

*Source: compiled by the authors.*

Considering the high dynamics of skull growth in children of the first two years of life, the analysis of the results of a five-month course of helmet therapy in a group of patients shows a positive trend towards correction of deformities and compliance of changes with physiological norms. There is a positive trend, which is consistent with the physiological growth standards of children of this age range. The increase in the anterior-posterior size from 129 mm to 142 mm fits within the age norm (120–140 mm for children under 2 years of age), which indicates that there is no negative effect of helmets on the natural growth of the skull. A similar situation is observed with the side size, which increased from 123 mm to 129 mm, which also corresponds to the standard indicators (120–130 mm). The key result of therapy is a significant reduction in the asymmetry between LA and RA from 9 mm to 3 mm. This not only digitally demonstrates the corrective effect of helmets, but is also clinically significant, since a difference of less than 5 mm is considered normal. Thus, helmets not only do not interfere with the growth of the skull, but also actively contribute to its harmonisation. An increase in head circumference from 41.5 cm to 43.5 cm is also an important marker of adequate development. Considering the age of patients at the time of the second measurement (approximately 7–8 months), this indicator correlates with the standard values of 42–44 cm for 6-month-olds. This suggests that helmet therapy does not inhibit brain development and does not interfere with the normal growth of the skull. However, despite the positive averages, it is important to keep in mind individual variability and the need for a personalised approach to each patient.

Thus, the study demonstrated that optical 3D scanning is an effective, safe and accurate method for diagnosing cranial defects in young children. The use of individually made corrective helmets based on the obtained 3D models significantly improved the shape and symmetry of the skull during five months of treatment. Most parents noted the easy adaptation of children to helmets and a high level of comfort when wearing them. The results of measurements after therapy indicate positive dynamics of skull growth, which correspond to age norms, and confirm the effectiveness of a personalised approach in correcting cranial deformities in children.

## 4. Discussion

The study confirmed the high effectiveness of individually manufactured corrective helmets in the treatment of cranial deformities in young children. The use of optical 3D scanning provided detailed and safe diagnostics, detecting even minor defects. During the five-month therapy, a significant improvement in the shape and symmetry of the skull was achieved, which is confirmed by objective measurements and positive growth dynamics corresponding to age norms. Parents noted the easy adaptation of children to wearing helmets and a high level of comfort, which emphasises the safety and acceptability of the method. Thus, the study demonstrates the feasibility and effectiveness of a personalised approach in correcting cranial deformities in children using corrective helmets.

As for the work of J. González-Santos et al. [29], whose study compared two treatments for positional plagiocephaly in infants: helmet therapy and physical therapy. 48 children aged 5 to 10 months with cranial deformities were studied, and the evaluation of the results was based on cranial vault asymmetry index (CVAI) and the Brunet-Lézine Developmental Scale. After 40 sessions, both methods showed a significant improvement in functional status, with no significant differences between them: CVAI decreased by 4.07% in the helmet group and 5.85% in the physical therapy group. As a result, the researchers note that such data indicate similar effectiveness of both methods, which allows doctors to recommend physical therapy as a less invasive and affordable option. Unlike the paper by J. González-Santos et al., the current study used optical 3D scanning for accurate diagnosis and custom manufacturing of corrective helmets, which significantly reduced skull asymmetry from 9 mm to 3 mm, which is a clinically significant result. In addition, this study considered the comfort and adaptation of the child to treatment, which increased the effectiveness and acceptability of the chosen method.

In turn, N. Rouhani et al. [30] focused on studying factors influencing parents’ willingness to follow orthopaedic treatment for skull remodelling in children with positional deformities. Data was collected through interviews with parents, which revealed the main barriers that families faced during treatment. Among them were transport difficulties, lack of insurance coverage for orthoses, the need for frequent visits to clinics, restrictions on physical contact with the child due to the use of a helmet, and negative attitudes of others. In contrast, the study also identified positive factors that contributed to treatment success, such as high parental motivation, support from healthcare professionals, positive experience interacting with an orthotist, aesthetic satisfaction with treatment outcomes, and stable support from family members. The findings highlight the importance of a comprehensive approach to supporting parents, which can increase their willingness to follow a treatment plan. Identified barriers, such as transportation problems, lack of insurance coverage, and social pressures, made it difficult to follow the treatment regimen. The current study, aware of these obstacles, focused on reducing the need for frequent clinic visits through the use of modern technologies. Optical 3D scanning and custom helmet manufacturing have increased parental autonomy and reduced logistical difficulties. Active interaction with parents and providing them with support also increased their motivation and compliance with the treatment plan, based on which it was possible to get the most positive result from therapy with corrective helmets.

A. di Chiara et al. [31] considered a conservative approach to the treatment of positional plagiocephaly in infants, which consists of the use of physical therapy and position correction. Treatment included exercises to strengthen the neck muscles and change the position of the head to avoid preference for a certain position and help to reduce deformities. The results of the study showed that physical therapy and position correction can be effective in treating mild to moderate forms of plagiocephaly, especially when treatment begins at an early stage (up to 4 months). The study also notes that exercise helps to avoid preference for one head position, which has a positive effect on correcting the shape of the skull. The advantages of this method, according to the researchers, include the absence of invasiveness, cost-effectiveness and accessibility for most families, since physical therapy does not require expensive equipment or special means, such as orthopaedic helmets. However, the effectiveness of physical therapy is highly dependent on the active participation of parents, which can be difficult due to time or resource constraints. In the current study, the use of corrective helmets reduced the burden on parents, as the helmet provides a constant corrective effect without the need for constant monitoring on their part, which makes this method more practical and effective, especially for families with limited resources.

P. Jeyaraj [32] analysed treatments for skull deformities that occur as a result of combat injuries, such as explosive injuries or shrapnel wounds. The researcher emphasised the need for a multidisciplinary approach to successfully restore the structural and functional integrity of the skull in victims. The paper described methods of primary and final restoration of skull injuries, focusing on the use of appropriate materials for cranioplasty and an individualised approach to each case. The results show that this approach contributes not only to the preservation of the anatomical structure of the skull, but also to the improvement of the psychological state of patients, returning them to the functionality and aesthetic appearance of the skull, which is important for rehabilitation and social adaptation. Although the researchers emphasise the importance of a multidisciplinary approach and individual selection of materials for cranioplasty, this approach is invasive and requires a highly qualified team of specialists. The current study suggests a non-invasive treatment for cranial deformities in young children that minimises risks and stress for patients. The use of corrective helmets is a safe and effective method of correction, which can be applied on a larger scale without the need for complex surgical interventions, but this approach will be relevant exclusively for children under 2 years of age, as physiological changes make it impossible to use this method of therapy after this age.

The study by A. Ciukszo et al. [33] was devoted to the use of cranial orthoses as a method of correction of cranial deformities in infants with positional plagiocephaly. The study showed that the best results are achieved when starting treatment before the 5th month of an infant’s life, when the skull bones are still quite flexible. The study notes that helmets can effectively correct the shape of the skull in cases where conservative methods, such as physical therapy or position correction, are insufficient. The method is considered safe and effective, especially for the treatment of severe deformities, which makes it popular among parents and medical professionals who want to quickly and safely solve the problem of skull asymmetry in children. This study is generally consistent with the current study, highlighting the efficacy and safety of using custom-made corrective helmets to correct cranial deformities in young children.

D.J. Caycedo et al. [34] analysed the surgical method of osteotomy using multiple rotational manipulations to treat simple craniosynostosis, a disease that occurs due to premature fusion of cranial sutures in children. The study included 20 cases of patients who underwent this procedure and showed successful results without complications in the postoperative period. After 90 and 180 days of follow-up, no clinical complications or repeated suture fusion were detected, which confirms the effectiveness of the procedure and its positive effect on the shape and functionality of the skull. Surgical correction allowed achieving a significant improvement in the shape of the skull, which contributed not only to functional recovery, but also to improving the aesthetic appearance, which is of great importance for the social and psychological comfort of patients. Although the results show the effectiveness of the procedure without complications, the method is invasive and requires specialised surgical intervention. The current study suggests an alternative, non-invasive approach to correcting cranial deformities that is safer and less stressful for the child. The use of corrective helmets avoids the risks associated with operations, and provides effective correction of the shape of the skull.

Thus, the study is marked by the innovative use of optical 3D scanning for accurate and safe diagnostics, which allows for the detection of even minimal defects. Custom-made corrective helmets provide a personalised approach to treatment, increasing its effectiveness and comfort for the child. Social and economic aspects are considered to reduce barriers to access to treatment and increase parental compliance. Objective evaluation of the results using quantitative measurements confirms significant improvements in the shape and symmetry of the skull. The combination of these factors suggests that the study not only addresses the gaps found in other studies, but also sets new standards for the diagnosis and treatment of cranial deformities in young children.

While the present study provides valuable insights into the effectiveness of orthotic therapy for cranial deformities, it is limited by the absence of long-term follow-up, which is crucial for assessing the recurrence or maintenance of results over time. Cranial remodelling, especially in young children, is a dynamic process, and it is essential to understand whether the improvements achieved through therapy are sustained as the child grows and the skull matures. Without long-term data, it remains unclear whether the positive outcomes observed during the study period are maintained or if there is a risk of relapse as the skull becomes less malleable with age.

Future research should include extended follow-up periods to assess the durability of the treatment outcomes. Long-term studies could provide important information on whether the improvements in cranial symmetry remain stable or if additional interventions are needed as the child develops. Furthermore, understanding the factors that contribute to the maintenance of results, such as the age at the start of treatment and the severity of the initial deformity, would offer valuable guidance for clinicians in determining optimal treatment timelines and post-therapy care. Conducting such studies will not only enhance our understanding of the long-term effectiveness of non-surgical treatments but also contribute to refining therapeutic strategies for cranial deformities.

## 5. Conclusions

In the course of the study, it was determined that individually manufactured corrective helmets are a highly effective means of non-surgical treatment of skull deformities in children under 2 years of age. The use of 3D optical scanning as the main diagnostic method allowed for accurate and safe assessment of the degree and nature of deformities, which contributed to the manufacture of helmets considering the anatomical features of each child. This approach provided personalised treatment, which had a positive impact on the effectiveness of therapy.

During the five-month therapy period, significant improvements in the shape and symmetry of the skull were observed in most patients. In particular, the asymmetry between the left and right anterior skull sizes decreased from an average of 9 mm to 3 mm, which corresponds to the age norm and indicates a significant alignment of the skull shape. Other craniometric parameters, such as anterior-posterior and lateral dimensions, also showed positive dynamics corresponding to the physiological growth standards of children of this age, which means that therapy not only corrects deformities, but also does not interfere with the natural development of the skull and brain.

An important qualitative indicator is the high satisfaction of parents with the results of treatment. Most parents noted the easy adaptation of children to wearing helmets and a high level of comfort during therapy. They also noted noticeable improvements in the shape of the child’s head, which highlights the practical value of the method and its positive impact on the quality of life of the family. This shows that the method is not only medically effective, but also acceptable and convenient in everyday life. The results obtained are important for the practice of paediatrics and paediatric neurology, as they offer a safe and effective method for correcting cranial deformities without surgery. This avoids possible risks associated with operations and provides comfortable conditions for the treatment of young children.

A limitation of the current study is a relatively small sample and a limited follow-up period. Future studies will compare the effectiveness of helmet therapy with other non-surgical treatments, such as kinesiotherapy and postural therapy, to determine the best approach.

## Figures and Tables

**Figure 1.**
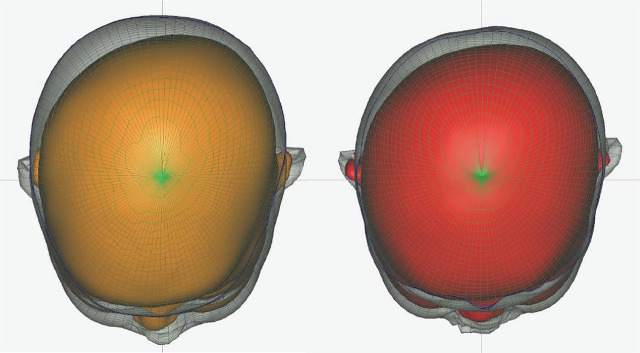
Three-dimensional reconstruction of the infant skull before treatment (axial view). The image demonstrates asymmetry in the occipital region with notable flattening on one side, characteristic of positional plagiocephaly.

**Figure 2.**
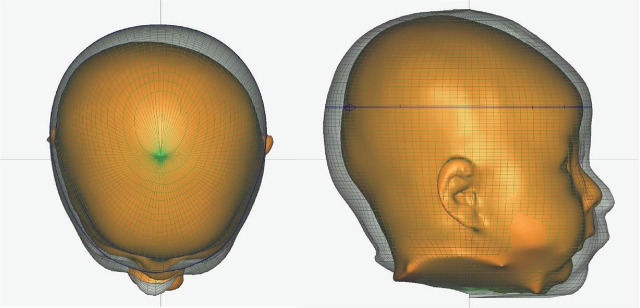
3D model of the skull before treatment (sagittal view). Visible deviation of cranial curvature and occipital flattening indicate a mild to moderate degree of deformity prior to helmet therapy.

**Figure 3.**
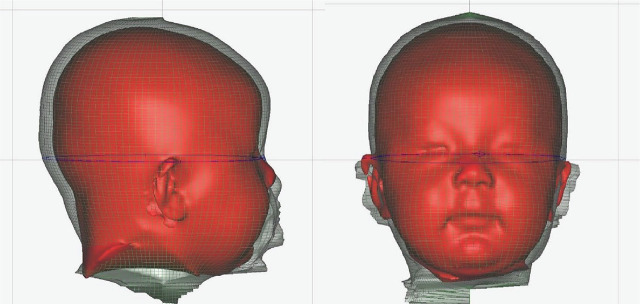
3D model of the skull before treatment (frontal view). The anterior asymmetry between left and right frontal bones highlights the lateral shift of cranial volume.

**Figure 4.**
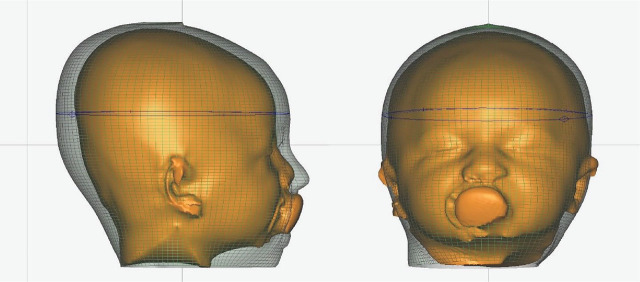
Color-coded deviation map of the skull surface before treatment. Warmer colors correspond to convex regions, and cooler tones to concave areas, illustrating a 9 mm difference between left and right frontal dimensions.

**Figure 5.**
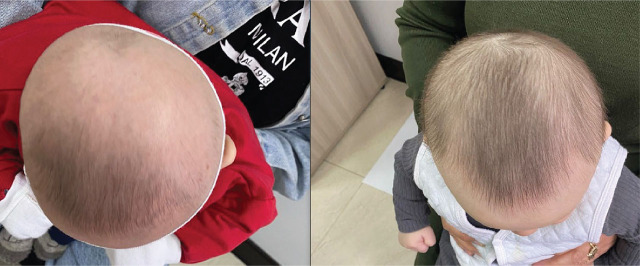
Three-dimensional reconstruction of the skull after 5 months of helmet therapy (axial view). The occipital region shows restored roundness and symmetry.

**Figure 6.**
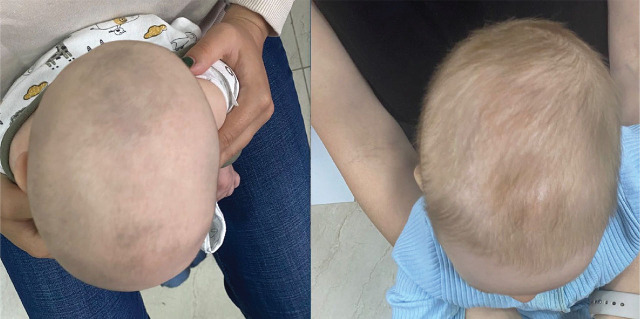
3D model of the skull after treatment (sagittal view). Improved cranial curvature and balanced anterior–posterior proportions are evident.

**Figure 7.**
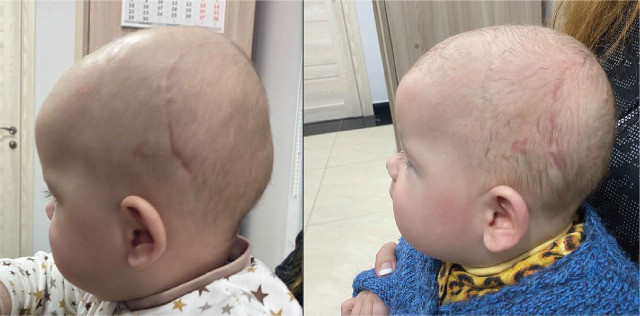
3D model of the skull after treatment (frontal view). The previous asymmetry between left and right frontal areas is no longer observable.

**Figure 8.**
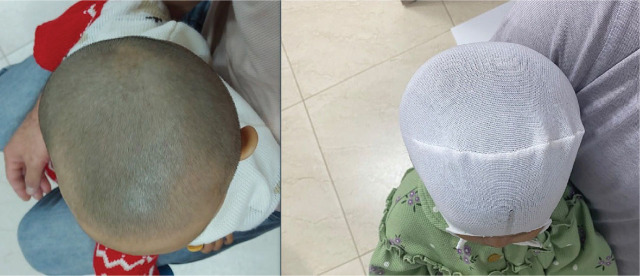
Comparative overlay of pre- and post-treatment 3D models. The reduction in lateral deviation and improved alignment of cranial contours confirm successful correction.

**Figure 9.**
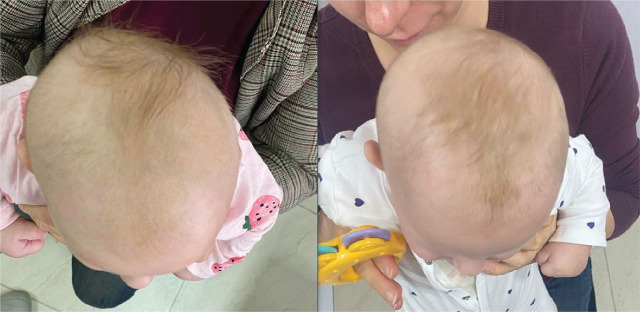
Color deviation map after therapy. The uniform green–yellow coloration indicates restoration of cranial symmetry with residual variation ≤3 mm.

**Figure 10.**
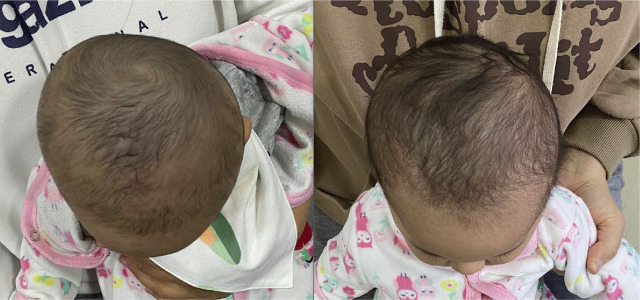
Quantitative analysis of craniometric parameters before and after treatment. The bar chart presents anterior–posterior, lateral, and circumferential measurements demonstrating improvement consistent with age norms.

**Figure 11.**
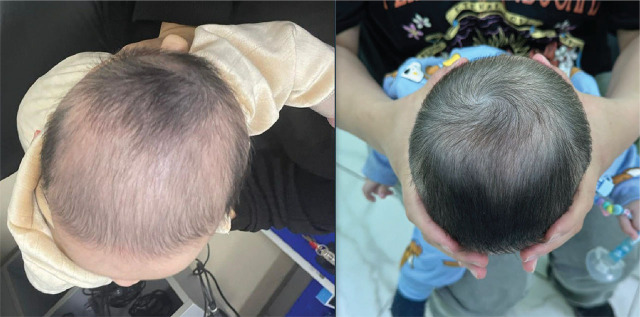
Photographic comparison of patients before and after helmet therapy. The visible normalization of head shape and cranial symmetry illustrates the clinical effectiveness and aesthetic outcome of orthotic treatment.
